# The Frequency of Rh Phenotype and Its Probable Genotype

**DOI:** 10.7759/cureus.25775

**Published:** 2022-06-09

**Authors:** Faryal Tariq, Javeria Ashfaq, Rehana Ahmed, Naveena Fatima, Yumna Ahmed, Munira Borhany

**Affiliations:** 1 Hematology, National Institute of Blood Diseases and Bone Marrow Transplantation, Karachi, PAK; 2 Research and Development, National Institute of Blood Diseases and Bone Marrow Transplantation, Karachi, PAK; 3 Radiation Oncology, Aga Khan University Hospital, Karachi, PAK

**Keywords:** alloimmunization, transfusion, blood group, probable genotype, phenotype, rh system

## Abstract

Aims and objectives: Our goal is to disseminate data on the distribution pattern of Rh antigen, its phenotypes, and the likely genotypes of these genetic variants in the Pakistani population.

Methodology: This study was a cross-sectional research project. Patients’ demographic statistics, such as age and gender, were gathered from their medical information. Blood group, disease, RhD, and other antigen frequency, phenotype, and probable genotype were considered variables. All blood samples were phenotyped for Rhesus antigens (D, C, c, E, and e), and the test was carried out using the tubing technique.

Results: According to gender distribution, most of the patients were males, with 131 frequencies (57.7%), while females had 42.35%. The most common phenotype was DCCee, with its probable genotype DCe/DCe (*R1 R1*) (34%), followed by DCcee, with probable genotype DCe/ce (*R1 r*) (29.1%); the least common phenotype was ddCcee, with its probable genotype Ce/ce (*r ' r*) (0.4%).

Conclusion: It is concluded that the DCCee phenotype was the most common with its probable genotype DCe/DCe, while the least common phenotype was ddCcee with its probable genotype Ce/ce.

## Introduction

The genotype is a patient’s actual genetic makeup, and the influence of genes can be clinically seen by the perceived consequence, which is characterized as the phenotype [1]. The Rhesus blood group system is highly polymorphic and immunogenic, particularly in humans [2]. Clinically, it remains the most essential and well-known blood group system [3]. In 1940, Landsteiner and Weiner introduced this Rh blood group system [4]. According to Weiner’s theory, Rh, Rh1, Rh2, Rhz, rh, rh1, rh2, and rhy are allele genes. He presented the hypothesis of a single locus with eight allele genes. The nomenclature of Weiner was complicated and not widely used [5]. Numerous recent reports from throughout the globe have added support to the argument that people vary genetically regarding the Rh blood type system, which consists of 49 antigens produced by genes on chromosome 1. The most significant are RhD, RhC, RhE, Rhc, and Rhe [6,7]. Alloimmunization and antibody production against the Rh or negligible genetic profile antigens, including Kell, MNSs, and Duffy, might happen in patients also after much blood categorizing and cross-matching. Several advanced economies have altered the existing cross-match policy and commenced sequencing all potential volunteers to produce a vast donor registry for future purposes and referrals [8-11]. Individuals who may necessitate multiple donations in the prospective are included in this group; other countries have made comprehensive phenotyping and complete cross-matching mandatory [12]. These techniques have substantially increased the cost of blood banking in wealthy countries, delaying its deployment in underdeveloped countries such as Pakistan. Given the high cost of comprehensive phenotyping, determined only in multi-transfused patients, Rh phenotypes can be crucial in decreasing alloimmunization and minimizing unfavorable consequences following transfusion [13-16]. There is minimal information on the gene frequencies of Rhesus blood in Pakistani studies. Our goal is to disseminate information on the Rh antigen distribution pattern, its phenotype, and the likely genotypes of these genetic variants in the Pakistani population. This research will also help produce local data for creating a donor panel, particularly for multi-transfused and alloimmunized patients.

## Materials and methods

The cross-sectional study was conducted at the National Institute of Blood Diseases and Bone Marrow Transplantation, Karachi, Pakistan, from 2019 to 2021 after getting approval from the Institutional Review Board Committee. A convenient sampling technique was used for the collection of data. There was a total of 62 subjects, as calculated using the following formula: 

$n=\ \frac{Z^21-\alpha/2^*\sigma^2}{d^2}$.

n is the calculated sample size, z1-∝/2 is the z score at a 1-∝/2 confidence level, σ is the estimated standard deviation, and “d” is the largest difference of the estimated mean that could be accepted in the research. Overall, 227 samples of non-transfused patients from the previous three months were collected. Patients’ demographic information, e.g., age and gender distribution, were obtained from their medical records. Blood group, disease, RhD, other antigen frequency, phenotype, and probable genotype were considered variables. Without molecular studies, there is no way to be sure of genotype, so we calculate probable genotype following the most common phenotype and haplotypes. In a plain sterilized vial, 3 mL of blood from each individual was collected for serological testing. The gel technique was used for the laboratory assay. Monoclonal blood group antisera, such as anti-A, anti-B, anti-AB, anti-H, and anti-A1, were used for forwarding ABO grouping, while 5% pooled cell suspension of A, B, and O cells were used for reverse grouping. Gel technology was also used when required. A blood specimen of 5 mL was taken at the time of presentation into a tube containing ethylenediaminetetraacetic acid (EDTA) for antigen typing. For Rh determinants, all plasma samples were phenotyped (D, C, c, E, and e). Rh-positive units were those who had met the standards for RhD antigen. RhD genotype was performed using both the tube technique and gel technologies, utilizing a monoclonal/polyclonal anti-D and a gel card. Except for antigen D, specific monoclonal antisera were employed to determine the status of the majority of large antigens of the Rh system, namely, antigens C, c, E, and e, and the analysis was carried out using the tubing technique. The microplate hemagglutination analysis measured red blood cells, particularly antisera, to detect antigen-antibody responses (hemagglutination). The direct agglutination of red cells determined the existence of an antigen with a specific solution. There was no agglutination to suggest that it was missing. All patients willing to participate with non-transfusion from the previous three months were included. There were no age restrictions. There are no biases found in our study. In addition, patients who were unwilling to participate and received a recent transfusion of red blood cells within three months were excluded from the study. The SPSS version 23.0 (IBM Corp., Armonk, NY, USA) was used for the analysis of the collected data. Frequencies were presented in the form of tables. Descriptive statistics have also been measured. The correlation between antigens, diagnosed disease, and patients’ blood groups has been calculated by applying Spearman’s rho correlation. The observed phenotype and its probable genotypic frequency were compared using the chi-square test with a (95% confidence interval) significance of 0.05.

## Results

Table 1 shows the frequency of the demographics of patients. According to gender distribution, most of the patients were males (n = 131, 57.7%), while females were 42.35% with a frequency of 96. There were more patients in the age group between one and 10 years (n = 136, 59.9%). Most of the patients have beta-thalassemia disease (BTD) (73.6%), while fewer patients have sickle cell disease (SCD) (2.2%), pure red cell aplasia (PRCA) (0.4%), and autoimmune hemolytic anemia (AIHA) (0.4%). In the blood groups of the population, most of the patients (35.2%) were O Rh (D)-positive. Other details are presented in Table 1.

**Table 1:** Frequency of the Demographic Characteristics of the Patients.

Variables	Frequencies (n = 227)	Percentage (%)
Gender		
Male	131	57.7
Female	96	42.3
Age groups (year)		
<1	33	14.5
1-10	136	59.9
11-20	33	14.5
21-30	12	5.3
>31	13	5.7
Diagnosed disease before blood transfusion		
Beta-thalassemia disease (BTD), including major, intermediate, and compound heterozygotes: E-beta-thalassemia and D-beta-thalassemia	167	73.6
Iron deficiency anemia (IDA)	36	15.9
Aplastic anemia (AA)	17	7.5
Sickle cell disease (SCD)	5	2.2
Pure red cell aplasia (PRCA)	1	0.4
Autoimmune hemolytic anemia (AIHA)	1	0.4
Blood groups of the population		
A Rh (D) positive	50	22
A Rh (D) negative	5	2.2
B Rh (D) positive	57	25.1
B Rh (D) negative	5	2.2
O RH (D) positive	80	35.2
O RH (D) negative	8	3.5
AB Rh (D) positive	18	7.9
AB Rh (D) negative	4	1.8

**Figure 1:**
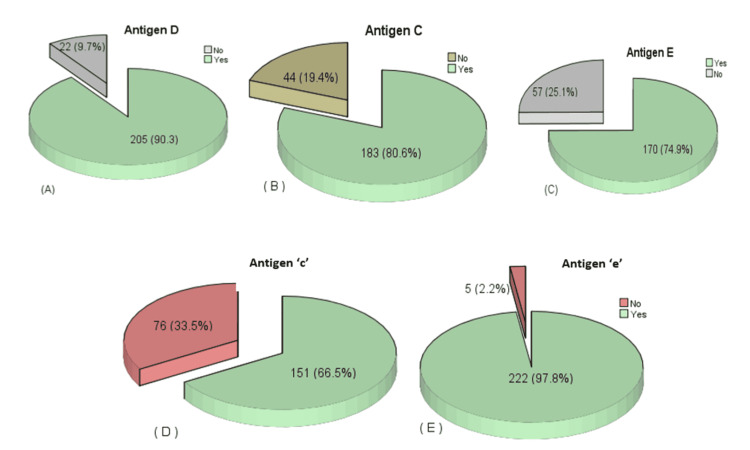
Graphical Explanation of the Frequency of Rh System Antigens: (A) Antigen D. (B) Antigen C. (C) Antigen E. (D) Antigen ‘c.’ (E) Antigen ‘e.’

Figure 1 shows Rh system antigens D, C, E, c, and e. According to which, antigen D is shown in 90.3% of the patients, and 9.7% are antigen D-negative. More details are given in Figure 1.

Table 2 shows the distribution pattern of the Rh phenotype and its probable genotype. In our study, the most common phenotype was DCCee, with its probable genotype DCe/DCe (R1 R1) and DCe/Ce (R1 r ') (34%), followed by DCcee, with probable genotype DCe/ce (R1 r), DCe/Dce (R1 r), and Dce/Ce (R0 r'), with 29.1%. The least common phenotype was ddCcee, with its probable genotype Ce/ce (r ' r) (0.4%). DCcEe (R1R2) is the third most common, with 16.7%. More details are presented in Table 2.

**Table 2:** Distribution Pattern of the Rh Phenotype and Its Probable Genotype.

		dccee	dCcee	Dccee	DccEe	DccEE	DCcee	DCcEe	DCCee	P-value
Genotype	dce/dce	21	0	0	0	0	0	0	0	0.000
	dCe/dce	0	1	0	0	0	0	0	0	
	Dce/dce	0	0	5	0	0	0	0	0	
	DcE/dce	0	0	0	15	0	0	0	0	
	DcE/DcE	0	0	0	0	4	0	0	0	
	DCe/dce	0	0	0	0	0	66	0	0	
	DCe/DcE	0	0	0	0	0	0	38	0	
	DCe/DCe	0	0	0	0	0	0	0	77	

## Discussion

In the study of Khan et al. [17], it was highlighted that a very high percentage of Rh blood group type occurs in Pakistani individuals. Patients’ age ranged from 1 to 60 years old, and the male/ratio was 57.7:42.3. The percentage of RhD antigen is observed to be approximately 90.90%-92% across Pakistan. In the study of Karim et al. [18], it was demonstrated that the percentage of e-antigen was determined to be 99%. In our research, e-antigen was the highest recorded at 97.8%. Daniels [19] highlighted that the trend of e-antigen being the highest is commonly observed across the globe, among various races. Thakral et al. [20] had also demonstrated that e-antigen is the least widely presented Rhesus antigen worldwide, with a prevalence of 29% among whites, 17.9% among Indians, and 22% among blacks. A comparable proportion of e-antigen at 19% was observed in Pakistan [18]. Our study corroborated the results from all these studies, with merely 74.9% of the blood donors and patients having e-antigen. According to the findings of the current research, the c-antigen was found in 80.6% of the donated blood. Our results are comparable to those, where it is noted that c-antigen is less frequent, for instance, among the Africans (27%) and also among the Europeans (68%) [19]. On the other hand, the proportion of c-antigen observed in another Pakistani study was higher than in our research, as it was 87% [18]. These higher proportions are comparable to those observed in China (93%) and India (89.5%) [21]. The proportion of c-antigen noted in our study was 66.5%; this was although on the higher side, yet it was comparable to another study from Pakistan [18], where the observed proportion of c-antigen was 57%. The proportion of the DCCee Rh phenotype noted in our study was 34%, and dCcee was least observed at 0.4% of the total population. These higher proportions are comparable to those observed in a previous study [22], with DCCee at 42% and Dccee in black at 44%. On the other hand, the proportion of DCe/dce probable genotype observed in another Pakistani study [18] was higher than our research, as it was 87%. The proportion of DCcee phenotype noted in our study was 29.1%, with the second-highest proportion. The least noted proportion of the ddCcee phenotype in the study was 0.4%. The proportion of DCcEe noted in our study was 16.7%. Our study corroborated the results from all these studies, with merely 0.4% of the blood donors having DCe/dce Rh probable genotype. A higher proportion of DCe/dce probable Rh genotype was noted at 34% in our study. The proportions of DCe/ce, DCe/Dce, and Dce/Ce genotypes are the second highest at 29.1%, while DCe/DcE (R1 R2), DCe/cE (R1 r ''), DCE/Dce (Rz R0), DCE/ce (Rz r), and DcE/Ce (R2 r ') genotypes were the third-highest at 16.7% in our study. Our results were consistent with the previous studies. According to Hassab et al. [22], the common Rh genotypes are R1R2, R1r", R2r', R0R0, R0r, RzR1, R1r", R2r', R1r, R1R0, R0r', R1R1, R1r', R0, R0r', R1R1, R1r', R0, R0r', R1R1, and R1r. According to Rahman et al. [23], the most common Rhesus genotype is CDe/cDE (R1 R2), which accounts for 39.75% of the population, while Rh blood genotypes cde/cde (rr) account for just 1.75%. Pakistan has a diverse ethnic mix. Hence, it is crucial to study and report pertinent information regarding the frequency distribution of the Rhesus phenotype of blood donors [23].

There are only a few types of research on Rh phenotype prevalence from Pakistan. This study assisted us in determining the Rh antigen preponderance in our community, allowing blood donors and transfusion centers to anticipate the formation of alloantibodies in patients who received a blood transfusion. This study will also help in finding antigen-matched blood for alloimmunized patients and preparing in-house panel cells using a directory.

The limitation of this study is its being a cross-sectional study with a convenience sampling technique using a newly developed questionnaire and scoring system. Score validation and nationwide longitudinal studies are required. The study’s limitations also included a lack of information on Rhesus blood in the Pakistani population and small sample size. Because this was a single-center study, we intend to undertake a multicenter investigation in the future. The study’s strength was the availability of direction to prevent alloimmunization in multi-transfused patients.

## Conclusions

It is concluded that the DCCee phenotype was common, with its probable genotype DCe/DCe (R1 R1), and the least common phenotype was ddCcee, with its probable genotype Ce/ce (r ' r). The most frequent antigen among the five antigens of the Rh system was RhD, while the least common was antigen c. The DCCee phenotype and DCe/dce probable genotype were the most common. It is also indicated that Rh phenotypic frequency distribution and its credible genotype research play a crucial role in population-based studies and medical-legal difficulties, including, more crucially, transfusion practices. In the Pakistani population, phenotype and presumed genotype demonstrated a high level of differences.
